# Rewriting Systems and the Modelling of Biological Systems

**DOI:** 10.1002/cfg.363

**Published:** 2004-02

**Authors:** Jean-Louis Giavitto, Grant Malcolm, Olivier Michel

**Affiliations:** 1 LaMI u.m.r. 8042 du CNRS Université d'Évry Val d'Essone — GENOPOLE Tour Évry-2 523 Place des Terrasses de l'Agora Évry 91000 France; 2 Department of Computer Science University of Liverpool Liverpool L69 7ZF UK

## Abstract

This paper gives a brief survey of the use of algebraic rewriting systems for modelling and simulating various biological processes, particularly at the cellular level.


**Comparative and Functional Genomics**
*Comp Funct Genom* 2004; **5**: 95–99.
Published online in Wiley InterScience (www.interscience.wiley.com). **DOI:** 10.1002/cfg.363

**Conference Review**

### Rewriting systems and the modelling of biological systems

Jean-Louis Giavitto[1], Grant Malcolm[2]* and Olivier Michel[1]
[1] *LaMI u.m.r. 8042 du CNRS, Université d'Évry Val d'Essone — GENOPOLE, Tour Évry-2, 523 Place des Terrasses de l'Agora, 91000 Évry, France*
[2] *Department of Computer Science, University of Liverpool, Liverpool L69 7ZF, UK*

**Correspondence to: Grant Malcolm, Department of Computer Science, University of Liverpool, Liverpool L69 7ZF, UK. E-mail: grant@csc.liv.ac.uk*

Received: 13 November 2003
Revised: 18 November 2003
Accepted: 25 November 2003

### Abstract

**This paper gives a brief survey of the use of algebraic rewriting systems for modelling and simulating various biological processes, particularly at the cellular level. Copyright © 2004 John Wiley & Sons, Ltd.**

**Keywords:** **computational models for cell simulation; dynamical systems with a dynamical structure; rewriting systems; simulation**

### Introduction

Computer systems are designed and built to meet some need in the real world: to maintain records of chains of amino acids or of personal finances, to visualize tomographic data or to send text messages, to fly planes or guide satellites. Any such system is useful only insofar as it records, simulates, predicts or helps to control some element of the behaviour of the real world (or, perhaps, of a virtual world). In this respect, computer systems are models of something, and designing and building such systems is tantamount to constructing a model of that thing.

Computer science has developed (or appropriated) many languages and tools to help build these models, and to relate different models that operate on different levels of abstraction. In this paper we give a survey of how a family of these languages, *rewriting systems*, have been used to model a variety of biological processes.

### Rewriting systems

The mechanics of rewriting systems are familiar to anyone who has done high-school maths: a term can be simplified by repeatedly replacing parts of the term (*subterms*) with other, equivalent, subterms, e.g:

$$1/2 \cdot 2/3 \cdot 3/4 \to 1/3 \cdot 3/4 \to 1/4$$

The 'cancellation' rule that is applied here is:

$$M/N \cdot N/P \to M/P,$$

where $M, N$ and $P$ are variables representing arbitrary numbers (although, presumably, $N$ and $P$ are not zero).

This cancellation rule is probably more familiar where the left- and right-hand sides are separated by an equality symbol ('='), rather than the arrow used here. In this arithmetic example, what is important is simplifying the original term in such a way that the resulting term denotes the same number as that denoted by the original term. If we think of these terms as being the *same thing* as the number they denote, then the end result of the process of simplification is exactly the same as where we started from. We could, however, think of the terms as being more or less complex representations of a particular number, and we could think of the simplification process as moving

Copyright © 2004 John Wiley & Sons, Ltd.

from a more complex representation to a less complex representation.

Computation is very often all about processes: things change, and move into different states, sometimes even in a non-deterministic way. The languages that computer scientists use to describe and create processes reflect this, e.g. the use of an arrow rather than an equality symbol in the example above.

Rewriting systems are just as simple as this example suggests: *terms* are built up from *constants* (such as, '0', '1', etc.) and *operations* (such as multiplication and division) and a number of *rules* (such as the cancellation rule above) describe how terms can be rewritten. An individual rewriting system specifies particular sets of constants, operations and rules; the mechanics of using the rules to rewrite terms is common to all rewriting systems. The example above can be seen as a model of numbers (or of terms denoting numbers), with the rules describing how entities in the model interact; the examples we survey below use rewriting systems to model biological processes, e.g. by having constants that represent proteins or molecules, operations that represent ways in which proteins and molecules can be brought together, and rules to describe the effects of their interactions.

The theory of rewriting systems (see e.g [3,4]) lies in algebra and logic, areas that have been extensively and successfully applied in almost every branch of science. A key result is that rewriting systems are *Turing complete* — every computable process can be described by a rewriting system. Moreover, using rules to transform terms is such a basic operation that there are many languages and tools (see e.g. [8,17]) that make rewriting systems powerful tools for describing, exploring and reasoning about models.

### Modelling biological systems

We give a brief and selective survey of research that uses rewriting systems to model or simulate dynamic biological systems. The simulation of any dynamic system by a rewriting system relies on:

- Representing the states of the dynamic system by expressions (terms built from the constants and operations).
- Expressing the evolutionary rules of the dynamic system as rewriting rules.

If this can be done, then the process of applying the rewriting rules to an expression $e$ corresponds to a possible trajectory of the dynamic system starting from the initial state $e$.

Finding appropriate constants, operations and rules is at the heart of building these computational models, and is a difficult task that requires insight and creativity. However, certain *kinds* of operation occur again and again, and give distinct properties to the rewriting systems that are built on top of them. Consider, for example, an operation that 'adds' proteins together: such an operation might form chains of proteins, as in DNA strands, or it might build a 'soup' of proteins that are not bound in a sequence, but can move about and interact with one another. Either of these alternatives can be built into a rewriting system by imposing certain rules on how the 'addition' operation behaves: 'associativity' in the former, giving rise to *string rewriting*; and 'commutativity' in the latter, giving rise to *multiset rewriting*. These two approaches are topological, in that they constrain the neighbourhood of the proteins that are added together (immediate neighbours in the sequence, in the first case, and any other protein in the 'soup' in the second).

We now look at both of these topological approaches, then at approaches to capturing more sophisticated topologic structures.

### String rewriting

String rewriting has been successfully applied in modelling plant development. Introduced in 1968 by Lindenmayer [16], the *L system* formalism is characterized by the parallel application of rewriting rules on strings representing a linear or a branching structure. The original L system formalism has been extended in many ways and a comprehensive review can be found in Prusinkiewicz [20,21]. A good example of its use that takes into account cellular interaction is the modelling of growth and heterocyst differentiation in *Anabaena*. This cyanobacterium grows in filaments of 100 cells or more. When starved for nitrogen, specialized cells called heterocysts differentiate from the photosynthetic vegetative cells at regular intervals along each filament. Heterocysts are anaerobic factories for nitrogen fixation; in them, the nitrogenase

Copyright © 2004 John Wiley & Sons, Ltd. 

enzyme complex is synthesized and the components of the oxygen-evolving photosystem II are turned off. Plant signals exert both positive and negative regulatory control on heterocyst differentiation. Wilcox *et al.* [23] have proposed an activator–inhibitor model of heterocyst differentiation where the (high) concentration of the activator triggers the heterocyst differentiation. The production of the activator is an autocatalytic reaction and also catalyses the production of the inhibitor. The inhibitor represses the activity of the activator when its concentration is high enough. The diffusion of the inhibitor to the neighbouring cells prevents neighbours becoming heterocysts and explains why heterocysts appear in a regularly spaced pattern in the filament. A computer simulation of this process [13] based on the use of parametric L systems [22] validates the model. This example is remarkable for at least two reasons: it shows the ability of this kind of discrete model to accommodate features usually handled in continuous formalisms (e.g. the modelling of diffusion) and also because it tackles a fundamental biological mechanism: a morphogenesis driven by a reaction–diffusion process taking place in a growing medium.

### Multiset rewriting

In a chemical solution, molecules move around and can interact with any other molecule. The state of the chemical solution can be modelled as a *multiset*, a set where an element is allowed to occur multiple times. We write $a \oplus b \oplus c \oplus b$ for a multiset containing elements (e.g. molecules) $a$, $b$ and $c$, where $b$ occurs twice. The operation $\oplus$ therefore builds a 'soup' of elements by 'adding' them together. Technically, we say that this addition operation is associative and commutative, which means that the elements can be written in any order: the soup $a \oplus b \oplus c \oplus b$ is the same as, for example, $b \oplus b \oplus c \oplus a$.

Once we have represented the state of a chemical solution as a multiset, it is then easy to formulate the chemical reaction rules as multiset rewriting rules, e.g:

$$r_1\colon a \oplus a \to a \oplus a \oplus b \qquad r_2\colon a \oplus b \to a \oplus b \oplus b$$
$$r_3\colon b \oplus b \to b \oplus b \oplus a$$

represent second-order catalytic reactions between two molecule types $a$ and $b$. For example, if reaction $r_1$ occurs in a state $a \oplus c \oplus a \oplus b$, then the result is a state $a \oplus c \oplus a \oplus b \oplus b$, one additional $b$ is produced. (Note that it does not matter that the two $a$'s were not side-by-side in the first state, because a multiset can be written in any order; this is just the same thing as applying the arithmetic cancellation rule to a term $1/3 \cdot 2/7 \cdot 3/5$.)

This abstract approach to chemistry is now recognized as an emerging field called *artificial chemistries* (see [5]) and embraces a wide variety of research, ranging from the study of the automated generation of combustion reactions [2] to the study of complex dynamic systems and self-organization in biological evolution [10].

Fisher *et al.* [9] proposed the use of rewriting systems to model cascades of protein interactions in signalling pathways. In this context, multisets provide a convenient way of making the participating proteins available for the individual reactions in the cascade. Later work by Eker *et al* [6,7] has produced some very sophisticated models of these pathways; however, the earlier work draws attention to the subtle role that so-called 'scaffold proteins' play in facilitating cascades and preventing cross-talk between pathways. These scaffold proteins can be seen as introducing interesting topological structure among the proteins that they bind; a kind of structure that is not, in itself, at odds with the multiset approach, but which suggests that more structured approaches to intracellular protein interactions, and other biological dynamic systems, would be a fruitful avenue of research.

### P systems

Several variations on multisets have been proposed to facilitate the representation of more sophisticated biological structure, e.g. one can 'nest' multisets one within another, so that the elements of the multiset can be both molecules and multisets (which may in turn contain both molecules and other multisets, and so on). This approach can be used to represent ecologies of cells and proteins, where the nested multisets represent cells, or even compartments, such as sites, within cells. Such nesting of multisets is developed in the domain of *P systems* [18,19]. This paradigm extends standard multiset rewriting by introducing the notion of 'membrane'. A membrane structure is a nesting of compartments represented, for example, by a Venn diagram without intersection and with a unique superset:

Copyright © 2004 John Wiley & Sons, Ltd.

the skin. Objects are placed in the regions defined by the membranes and evolve following various transformations: an object can evolve into another object, can pass through a membrane or dissolve its containing membrane. In the initial definition of the P systems, each region defined by a membrane corresponds to a multiset of atomic objects which can evolve following some evolutionary rules. The membrane structure enables the specification of some localization of the processes and a region can be equipped with various computational mechanisms: multiset rewriting, string rewriting, splicing systems, etc. An example of this approach, modelling a spatially distributed biochemical network, is given in Giavitto and Michel [12].

P-systems represent a particularly well-developed approach to integrating complex topological structures into rewriting systems; other approaches, as well as the issues concerning dynamically changing topological structures, are discussed in Giavitto and Michel [12].

### Conclusion

The examples above indicate that rewriting systems and tools such as the languages Maude [17] and ELAN [8] can be effectively used in modelling biological systems. The speed of such tools also makes them particularly effective in simulating and exploring the models that are built.

By combining and structuring multiset and string rewriting, we can extend the applicability of these formalisms. Applications of such extensions at the genetic level include DNA computing [1] and *splicing systems*, a language-theoretic model of DNA recombination that allows the study of the generative power of general recombination and of sets of enzymatic activities [14,15]. However, the need to represent more structured organizations motivates further extensions of rewriting (see e.g. [3,11].

To conclude, we want to emphasize the versatile nature of rewriting formalisms. Models can be qualitative or quantitative. They also support an individual-based simulation style by computing the global consequences (the derivations) of the local interactions (the rules) between the system entities. This versatility should be a big advantage in biological applications.

### References

1. Adleman LM. 1994. Molecular computation of solutions to combinatorial problems. *Science* **266**(5187): 1021–1024.
2. Bournez O, Côme G-M, Valérie Conraud HK, Ibanescu L. 2003. A rule-based approach for automated generation of kinetic chemical mechanisms. In *14th International Conference on Rewriting Techniques and Applications (RTA '03)*, vol 2706 of *Lecture Notes in Computer Science*, Nieuwenhius R (ed.). Springer: Heidelberg; 30–45.
3. Brown R, Heyworth A. 2000. Using rewriting systems to compute left kan extensions and induced actions of categories. *J Symbol Comput* **29**(1): 5–31.
4. Dershowitz N, Jouannaud J-P. 1990. Rewrite systems. In *Handbook of Theoretical Computer Science*, vol B, Elsevier Science: Amsterdam; 244–320.
5. Dittrich P, Ziegler J, Banzhaf W. 2001. Artificial chemistries — a review. *Artificial Life* **7**(3): 225–275.
6. Eker S, Knapp M, Laderoute K, Lincoln P, Talcott C. 2002a. Pathway logic: executable models of biological networks. In *Fourth International Workshop on Rewriting Logic and Its Applications (WRLA '2002)*, vol 71 of *Electronic Notes in Theoretical Computer Science*, Gradducci F, Montanari U (eds). Elsevier: Amsterdam.
7. Eker S, Knapp M, Laderoute K, *et al*. 2002b. Pathway logic: symbolic analysis of biological signaling. In *Proceedings of the Pacific Symposium on Biocomputing*, Altman RB, Danker AK, Hunter L, Lauderdale K, Klein TE (eds). World Scientific: New Jersey USA; 400–412.
8. ELAN Home Page. 2002. **http://www.loria.fr/equipes/protheo/SOFTWARES/ELAN/**.
9. Fisher M, Malcolm G, Paton R. 2000. Spatiological processes in intracellular signalling. *BioSystems* **55**: 83–92.
10. Fontana W, Buss L. 1994. The arrival of the fittest: toward a theory of biological organization. *Bull Math Biol* **56**: 1–64.
11. Giavitto J-L, Michel O. 2002. The topological structures of membrane computing. *Fundamenta Informaticae* **49**: 107–129.
12. Giavitto J-L, Michel O. 2003. Modeling the topological organization of cellular processes. *BioSystems* **70**(2): 149–163.
13. Hammel M, Prusinkiewicz P. 1996. Visualization of developmental processes by extrusion in space-time. In *Proceedings of Graphics Interface '96*, Davis WA, Bartels R (eds). Canadian Human — Computer Communications Society: Toronto, Canada; 246–258.
14. Head T. 1987. Formal language theory and DNA: an analysis of the generative capacity of specific recombinant behaviors. *Bull Math Biol* **49**: 737–759.
15. Head T. 1992. *Lindenmayer Systems: Impacts on Theoretical Computer Science, Computer Graphics, and Developmental Biology*, Springer-Verlag: Berlin; 371–383; Also appears in *Nanobiology* 1992. **1**: 335–342.
16. Lindenmayer A. 1968. Mathematical models for cellular interaction in development, Parts I and II. *J Theoret Biol* **18**: 280–315.
17. Maude Home Page. 2002. **http://maude.csl.sri.com/**.
18. Paun G. 1998. Computing with membranes. Technical Report TUCS-TR-208, Turku Centre for Computer Science.
19. Paun G. 2001. From cells to computers: computing with membranes (P systems). *Biosystem* **59**(3): 139–158.

Copyright © 2004 John Wiley & Sons, Ltd. 

20. Prusinkiewicz P. 1998. Modeling of spatial structure and development of plants: a review. *Scientia Horticulturae* **74**: 113–149.
21. Prusinkiewicz P. 1999. A look at the visual modeling of plants using L-systems. *Agronomie* **19**: 211–224.
22. Prusinkiewicz P, Hanan J. 1990. Visualization of botanical structures and processes using parametric L-systems. In *Scientific Visualization and Graphics Simulation*, Thalmann D (ed.). Wiley: Chichester; 183–201.
23. Wilcox M, Mitchison GJ, Smith RJ. 1973. Pattern formation in the blue-green alga, *Anabaena*. I. Basic mechanisms. *J Cell Sci* **12**: 707–723.

Copyright © 2004 John Wiley & Sons, Ltd. 
